# Enhancing current-induced torques by abutting additional spin polarizer layer to nonmagnetic metal layer

**DOI:** 10.1038/srep45669

**Published:** 2017-04-04

**Authors:** Gyungchoon Go, Kyung-Jin Lee, Young Keun Kim

**Affiliations:** 1 Department of Materials Science and Engineering, Korea University, Seoul 02841, Korea; 2 KU-KIST Graduate School of Converging Science and Technology, Korea University, Seoul 02841, Korea

## Abstract

Recently, the switching of a perpendicularly magnetized ferromagnet (FM) by injecting an in-plane current into an attached non-magnet (NM) has become of emerging technological interest. This magnetization switching is attributed to the spin-orbit torque (SOT) originating from the strong spin-orbit coupling of the NM layer. However, the switching efficiency of the NM/FM structure itself may be insufficient for practical use, as for example, in spin transfer torque (STT)-based magnetic random access memory (MRAM) devices. Here we investigate spin torque in an NM/FM structure with an additional spin polarizer (SP) layer abutted to the NM layer. In addition to the SOT contribution, a spin-polarized current from the SP layer creates an extra spin chemical potential difference at the NM/FM interface and gives rise to a STT on the FM layer. We show that, using typical parameters including device width, thickness, spin diffusion length, and the spin Hall angle, the spin torque from the SP layer can be much larger than that from the spin Hall effect (SHE) of the NM.

As the use of mobile and IoT (internet of things) electronic devices increases, research on the development of devices with low power, small sizes, high speeds, and nonvolatility has become increasingly popular. Recently, the low energy consumption of magnetic random access memory (MRAM) devices has drawn considerable interest. Recent studies demonstrated that an in-plane current can manipulate the out-of-plane magnetization of the non-magnet (NM)/ferromagnet (FM) heterostructure via spin-orbit toque (SOT), which is caused by the bulk spin Hall effect (SHE) of a NM^1^,^2^,^3^,^4^ or the Rashba effect of a NM/FM interface^5^,^6^,^7^,^8^,^9^. The SOT switching mechanism provides not only rapid switching (over about few nanoseconds)^10^ but also device stability. However, there are a few challenges to be overcome in harnessing the SOT switching mechanism. One of biggest challenges facing the use of SOT devices in technological applications is the large switching current^11^. Theoretical^10^,^12^,^13^ and experimental^14^,^15^ studies report the switching current density required for SOT switching is much larger than is required for conventional spin-transfer-torque switching.

When injecting an in-plane current, the spin-orbit coupling (SOC) effect creates spin accumulation at the NM/FM interface and gives rise to torque on the FM layer. Therefore, one possible way to provide a suitable device structure that reduces the switching current is to increase spin accumulation at the NM/FM interface. For this purpose, we devise a device structure in which a ferromagnetic spin polarizer (SP) layer is abutted to a NM layer that can increase spin accumulation at the NM/FM interface. By properly adjusting the magnetization direction of the SP layer, the spin accumulation from the SP layer can combine additively to that due to the SOC effect. Thus, the additional spin accumulation from the SP layer is expected to enhance the spin torque acting on the FM layer.

In this study, we theoretically analyse the SP layer’s effect on the NM/FM heterostructure. The device geometry is illustrated in Fig. 1 which resembles a FM free layer (FL) portion of a magnetic tunnel junction (MTJ). Figure 1(a) shows a three-dimensional schematic view of the device structure with an FM (FL) with perpendicular magnetic anisotropy (PMA), whereas Fig. 1(b) shows a calculation domain. The current is injected in the *x*-direction, which induces the SOT to switch the magnetization of the FL. Unlike the usual NM/FM bilayer structure, there is one additional fixed FM layer (SP) beside the NM layer, in which the magnetization direction is set to the *y*-direction. In our theoretical model, as an approximation, we divide the device geometry into a combined sub-structure of the SP/NM (*x*-direction) and NM/FL (*z*-direction) layers and treat them separately.

## Model Calculation Methods

First, we calculate the spin accumulation for the SP/NM sub-structure. For simplicity, here we ignore the upper FM layer (FL) (the interface scattering at the SP/NM interface is also neglected). By solving the one-dimensional drift-diffusion equation^16^, one obtains the *y*-component of the spin accumulation in the NM layer as follows.


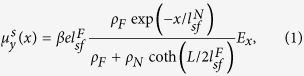


where *ρ*_*F*_ and *ρ*_*N*_ are the resistivities of SP and NM layers, *L* is the length (or width) of the fixed FM layer (SP), 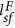 and 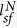 are the spin-flip diffusion lengths of the FM and NM layers, respectively, *E*_*x*_ is the applied electric field in the *x*-direction, and *β* is the spin polarization of the SP layer (−1 ≤ *β* ≤ 1). Here *x* = *0* is chosen as the SP/NM interface.

Next, we focus on the NM/FL sub-structure. When we apply the external electric field in the *x*-direction, in the NM layer, the charge and spin current densities flowing in the *z*-direction are given by ref. 17


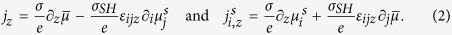


where *σ* is the conductivity of NM layer, *σ*_*SH*_ is the spin Hall conductivity of NM layer, and *ε*_*ijz*_ is the Levi-Civita symbol. Note that the usual definition of the spin current is 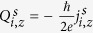, where the subscripts *i* and *z* stand for the spin orientation and the flow directions of the spin current, respectively. For the FL, the equations for the charge and spin currents are as follows.


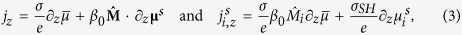


where 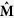 is the unit vector along the magnetization and *β*_0_ is the spin polarization of the FL.

The SP layer provides additional spin accumulation to the NM layer, which decays exponentially in the *x*-direction. Because it is difficult to obtain an analytical solution for the two-dimensional spin diffusion equation, as an approximation, we take an ansatz for the spin accumulation in the NM layer by simply adding an average spin accumulation from the SP layer (Equation (1)) to the solution of the diffusion equation in the *z*-direction. This gives


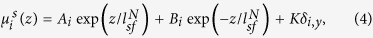


where we use an approximation 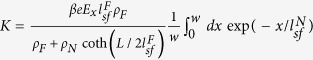, and *w* is the width of the structure. From Eqs (1) and (2), we obtain the averaged current flowing in *z*-direction 
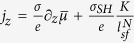
. As in ref. 18, we assume that the sample is infinite in the *y*-direction and sufficiently thin in the *z*-direction. In the steady state (*j*_*x*_ = constant, *j*_*z*_ = 0), the continuity equation ∇ · **j** = ∂_***z***_ *j*_*z*_ = 0 leads to 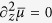. The solution of this equation is 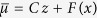, where *C* is a constant and *F(x*) represents the external electric field contribution, *F(x*) ∝ *eE*_*x*_*x*. Note that the current flowing in the *z*-direction is zero, *j*_*z*_ = 0 (this condition practically holds for an usual three terminal device including a magnetic tunnelling junction which has large resistance in z-direction), thus giving


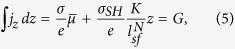


which results in


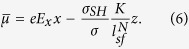


Using Eqs (2), (4), and (6), we obtain


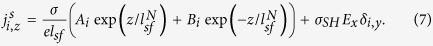


At the NM/FL interface, the charge and spin currents are given by refs 17,19, 20, 21


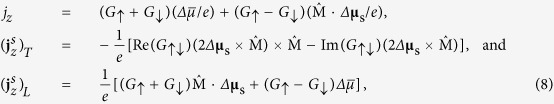


where the subscripts *T* and *L* represent the transverse and longitudinal components, *G*_↑_(*G*_↓_) is the interface conductivity of majority (minority) spin, *G*_↑↓_ is the mixing conductance, and 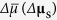 is the charge (spin) chemical potential drop over the interface. As in ref. 17, we assume that the spin dephasing length in the FL is very short, so that 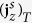 is fully absorbed at the NM/FL interface. Then, the spin torque is written as


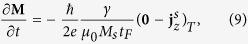


where *γ* is the gyromagnetic ratio and *M*_*s*_ is the magnetization per unit volume. The spin torque can then be rewritten in terms of damping-like and field-like torques as follows.


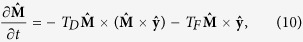


where *T*_*D*_(*T*_*F*_) is the coefficient of damping-like (field-like) spin torque.

Solving the bulk equations described above with the boundary conditions *j*_*z*_ = 0 and 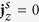 at the upper surface of the FL and lower surfaces of the NM (*z* = *t*_*F*_ and *z* = −*t*_*N*_), we obtain


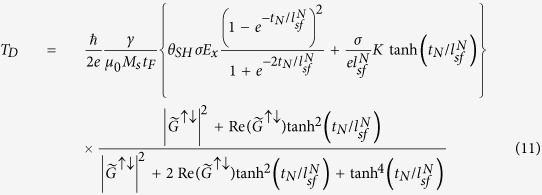


and


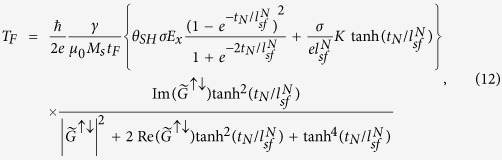


where *θ*_*SH*_ = *σ*_*SH*_/*σ* and 
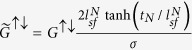
. In Eqs (11) and (12), the spin torques, which are localized at the interface of the NM/FL, are averaged over the FL layer’s thickness *t*_*F*_. The first term of each torque corresponds to the spin Hall contribution without the SP layer, which was computed by Haney *et al*.^17^. The spin polarizer effects are reflected in the second term. Because the spins in the NM layer are partially polarized by the SP layer, even without the SHE, a nonzero spin chemical potential drop at the NM/FL interface is induced that produces a spin torque. We note that the spin accumulation caused by the SP layer exerts a torque on the FL even without a direct current-flow perpendicular to the interface. This kind of spin torque has been experimentally observed in non-local geometry^22^ and also has been shown as a lateral spin torque caused by inhomogeneous magnetization^21^,^23^,^24^.

## Results

**Figure 2:**
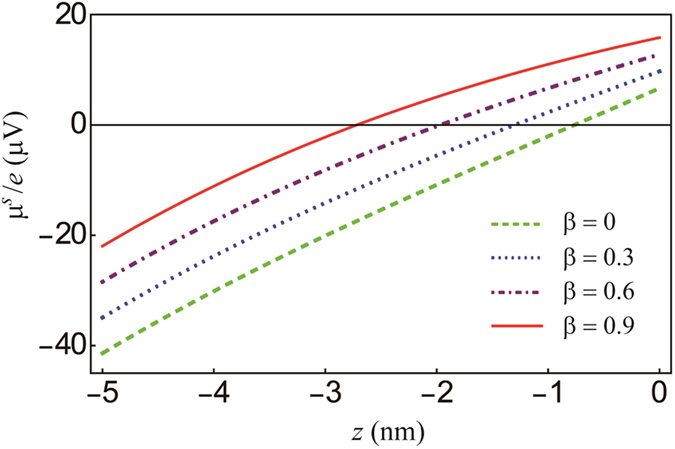
The spin accumulation 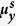 of the NM with different spin polarizations, *β* . As the spin polarization increases, the spin accumulation at the NM/FL interface (*z* = 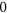) increases. The typical parameters *θ*_*SH*_ = 0.3, *σ*_*N*_ = 5 × 10^6^ m^−1^Ω^−1^, *σ*_*F*_ = 5 × 10^6^ m^−1^Ω^−1^, 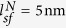, 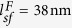, *L* = 20 nm, *t*_*N*_ = 5 nm, *t*_*F*_(*FL*) = 1.5 nm, *M*_*S*_ = 1.0 MA/m, 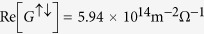, 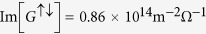, and *j*_*x*_ = 10^11^ A/m^2^ are used.

**Figure 3:**
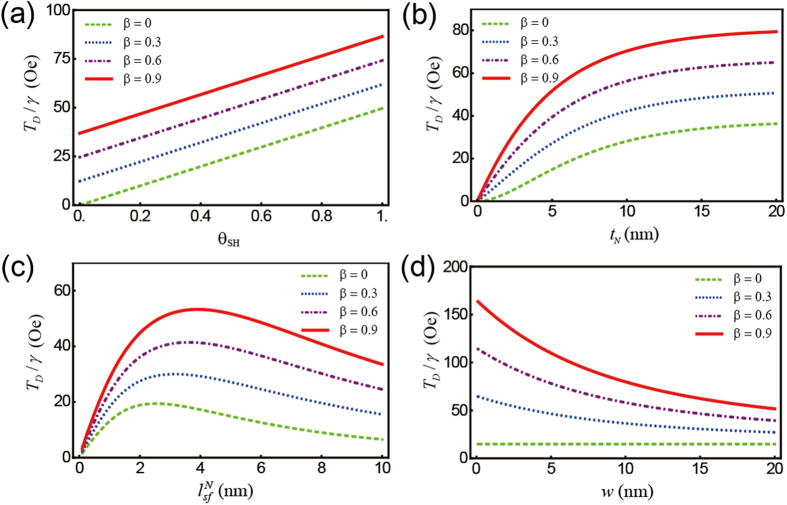
Damping-like (DL) component of the spin torques on the FL (1.5 nm) as a function of various parameters. (**a**) spin Hall angle *θ*_*SH*_, (**b**) NM thickness *t*_*N*_ (**c**) spin diffusion length 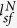, and (**d**) NM width *w* with different spin polarization factors, *β*. For each plot, except for the varying parameter (domain), a set of parameters including *θ*_*SH*_ = 0.3, *t*_*N*_ = 5 nm, 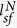 = 5 nm, and *w* = 20 nm is chosen. In all plots, the spin polarization dramatically enhances the spin torques.

The resultant spin torques are shown in Figs 2 and 3. We use the following parameters of Co (SP) and Pt (NM): *σ*_*F*_ = 5 × 10^6^ m^−1^Ω^−1^, 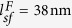
25, *σ*_*N*_ = 5 × 10^6^ m^−1^Ω^−1^, 
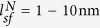
26, 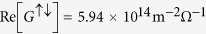, and 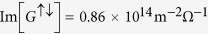
17. The applied electric field in the NM is chosen as *E*_*x*_ = 2 × 10^4^ V/m so that the charge current density in the *x*-direction is approximately *σ*_*N*_*E*_*x*_ ≈ 10^11^ A/m^2^. Other parameters are chosen as follows: magnetization of the FL is *M*_*S*_ = 1.0 MA/m, the length (or width) of SP layer is *L* = 20 nm, the spin Hall angle is *θ*_*SH*_ = 0–0.9, and the spin polarization is *β* = 0–1.0.

**Figure 4:**
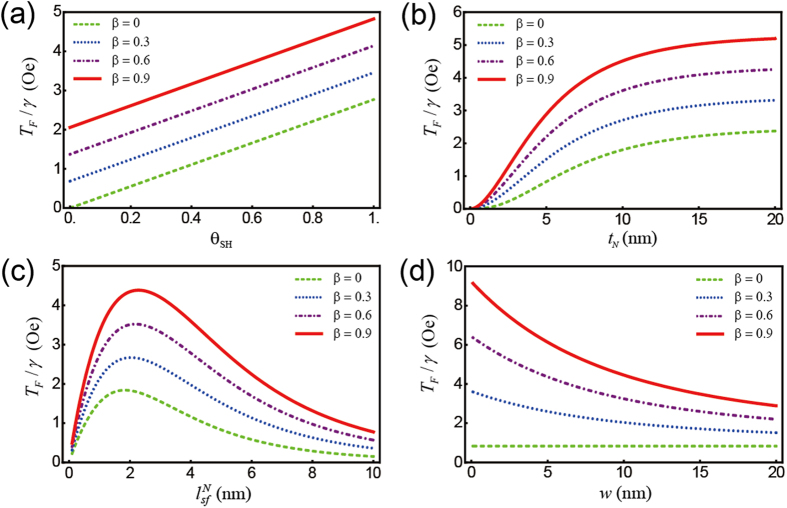
Field-like (FL) component of the spin torques on the FL (1.5 nm) as a function of various parameters. (**a**) spin Hall angle *θ*_*SH*_, (**b**) NM thickness *t*_*N*_ (**c**) spin diffusion length 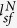, and (**d**) NM width *w* with different spin polarization factors, *β*. For each plot, except for the varying parameter (domain), a set of parameters including *θ*_*SH*_ = 0.3, *t*_*N*_ = 5 nm, 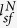 = 5 nm, and *w* = 20 nm is chosen. In all plots, the spin polarization dramatically enhances the spin torques.

Figure 2 shows the *y*-component spin accumulation 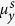 in the NM of the NM (5 nm)/FL (1.5 nm) structure for *θ*_*SH*_ = 0.3. Note that the net amount of spin accumulation at the upper edge (*z* = 0) significantly increases as *β* increases, which in turn enhances the spin torques in Eqs. (11) and (12). The dependence of the four parameters (*θ*_*SH*_, *t*_*N*_, 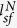, and *w*) on the damping-like spin torques *T*_*D*_ is summarized in Fig. 3. As shown in Fig. 3(a), the resultant spin torques increase with increasing *θ*_*SH*_. The dependence of *T*_*D*_ on *t*_*N*_ and 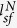 in Eq. (11) is complicated. In our parameterization, the resulting spin torques increase with increasing *t*_*N*_ (Fig. 3(b)) and have maximum values with varying 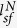 (Fig. 3(c)). Because the additional spin torque from the spin polarization decays along the *x*-direction, for nonzero *β*, the spin torque decreases with increasing NM width *w* (blue and red lines of Fig. 3(d)). For all plots, the spin polarization dramatically enhances the spin torques. We note the field-like torques *T*_*F*_ depicted in Fig. 4 are an order of magnitude smaller than the damping-like spin torques *T*_*D*_ because, in our parameterization, 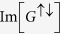 is smaller than 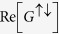, and the dependence of the four parameters on both torques is similar.

We propose that several other device geometries may also improve the switching performance. For example, another SP layer may be abutted on the other side (right side) of the NM. Because of spin diffusion, the magnitudes of spin torques from the SP layer decay along the *x*-direction; however, the decrease of the SP-layer effect would be compensated by another SP layer (oppositely magnetized) on the other side of the NM. In addition, by tilting the magnetization to *z*-direction, the proposed device structure may allow field-free switching. We note that conventional SOT switching requires an additional in-plane magnetic field for deterministic switching, which is not suitable for device engineering. Recently several reports have resolved this problem by breaking structural inversion symmetry^27^,^28^ or exchange bias from an antiferromagnetic layer^29^,^30^,^31^,^32^.

## Discussion

We have investigated the effects of both the SP layer and the spin Hall effect on the spin torque acting on the magnetization of the FM layer in an NM/FM heterostructure. Because of SP layer, the conduction electrons in the NM are partially polarized and provide an additional spin chemical potential change at the NM/FL interface. In our device structure, therefore, the SP layer was an additional source of spin torque. We also investigated the dependence of spin torque on parameters including *θ*_*SH*_, *t*_*N*_, 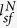, and *w*. Note that the resultant spin torques are maximized by minimizing the width of the sample, which can be beneficial for ultra-dense memory applications. Additionally, we have shown that, in typical parameterization, the spin torque from the SP layer is more effective than that from the spin Hall effect. Thus, the presence of the SP layer can significantly enhance the spin torque. With respect to the switching efficiency, we believe that our result provides insights into possible practical applications of STT-MRAM devices, which employ the current in-plane geometry.

We would like to note that the proposed structure can be applied to an MTJ utilizing standardized microelectronic fabrication technologies. For example, when a bottom-pinned MTJ (an FL on the top in the MTJ) is considered, an NM conductor can be patterned after film deposition, and then followed by SP layer deposition, patterning and field setting. Nevertheless, process development effort is necessary to achieve precise dimensional control.

## Additional Information

**How to cite this article:** Go, G. *et al*. Enhancing current-induced torques by abutting additional spin polarizer layer to nonmagnetic metal layer. *Sci. Rep.* **7**, 45669; doi: 10.1038/srep45669 (2017).

**Publisher's note:** Springer Nature remains neutral with regard to jurisdictional claims in published maps and institutional affiliations.

## Figures and Tables

**Figure 1:**
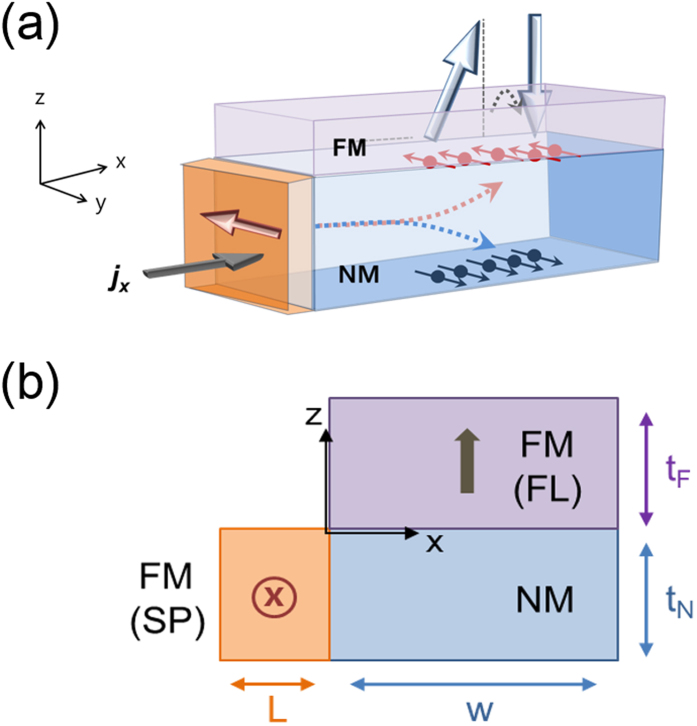
Schematic diagrams representing the proposed concept. (**a**) The NM/FM bilayer structure where an additional FM (SP) layer is abutted to the NM layer, and (**b**) the geometry of the structure considered for model calculation in this study. Note that the spin accumulation in the NM layer decays along the *x*-direction due to the presence of the SP layer as represented by the dotted line.
